# The persistent effect of acute psychosocial stress on heart rate variability

**DOI:** 10.1186/s43044-019-0009-z

**Published:** 2019-09-11

**Authors:** Alireza Mohammadi, Asgar Emamgoli, Marjan Shirinkalam, Golam Hossein Meftahi, Keyvan Yagoobi, Boshra Hatef

**Affiliations:** 1 Neuroscience Research Center, Baqiyatallah University of Medical Science, Mollasadra, Tehran, Iran; 2 American Liberty University, Newport Beach, CA USA; 3 Traditional and Complementary Medicine University of Iran and Armenia, Physiology Department, Islamic Azad University (IAUPS), Tehran, Iran

**Keywords:** Stress, Cortisol, HRV, Non-linear, Gender

## Abstract

**Background:**

As stress occurs repetitively every day, the biological modifiers should also have enough time to restore the normal state of hemostasis; otherwise, chronic stress would be anticipated. The aim of the present study was to examine the persistence of stress based on subjective emotion, salivary cortisol, and linear and non-linear features of heart rate variability (HRV) in both genders.

**Methods:**

Thirty-three healthy young volunteers (23 men and 10 women) participating in this study were exposed to the Trier Social Stress Test (TSST). Moreover, the emotional visual analog scale (EVAS), salivary cortisol, and ECG recording in the rest state were taken before and after TSST as well as 20 min after recovery.

**Results:**

According to the results of the two-way mixed model ANOVA, all volunteers showed a significant increase in EVAS after TSST which was restored to the baseline state after recovery. Notably, the women’s base of cortisol was significantly higher than men and the standard range of kit. Cortisol elevation was only observed in the men, and the significant increase of LF/HF ratio was observed in the women, while both did not retain to the baseline after recovery. The SD1 of Poincaré plot and spectral entropy decreased after stress in both genders. Moreover, there was a significant negative correlation between baseline level of cortisol and its elevation due to stress and some features of HRV.

**Conclusion:**

The base of cortisol played a critical role in modifying the physiological response to stress. In addition, after recovery, no stressful emotion remained, while the non-linear features of HRV did not return to baseline.

## Background

Stress activates two axes concurrently, hypothalamus-pituitary-adrenal (HPA) and sympathetic-adrenal medullary (SAM). The activation of HPA leads to the increase of cortisol in body fluids, and the activation of SAM increases the heart rate [37]. Acute psychosocial stress is a valid method of stress induction in human, which is followed by the assessment of the psychological, electrophysiological, and biochemistry indices [19]. The Triet Social Stress Test (TSST) as a model of psychological acute stress is a real condition that happens several times in a lifespan.

Several factors might affect the elevation of cortisol occurring through the activation of HPA axis during and after acute stress [8]. Some studies demonstrated that women are less sensitive than men especially in the follicular phase or contraceptive use [6, 26, 32]. According to other studies, individuals under stress can be divided into responder and non-responder to stress based on the change of cortisol. In fact, the non-responder has more medial prefrontal cortex (mPFC) activity than the responder group [27, 36]. On the other hand, Takahashi et al. [30] demonstrated that the increase of cortisol has a negative correlation with pretest cortisol and positive correlation with trait anxiety that needs more research [30]. However, the reason of responding or not responding to stress due to the change of cortisol is not yet clear.

The increase of heart rate (HR), LF, and ratio of LF/HF of heart rate variability (HRV) and decrease of RR mean of HRV, SD of Poincaré plot, and spectral entropy feature as the signs of SAM activity have been observed during TSST [4]. Although they immediately return to the baseline, the salivary cortisol level does not return to the rest condition even after half an hour [12]. The salience network activity gets strengthened during the acute stress phase; however, it is subsequently relocated by the executive control network to return to homeostasis by reversing this balance. This process takes a long time, about an hour [13]. Thus, whenever a participant reports no harmful feelings after stress, some biological markers show that they have not yet returned to the normal state, even after recovery [12, 32]. It should be noted that the findings showing that the cardiovascular system immediately returns to the baseline after stress may have ambiguity and need more assessments. In fact, if the increase of low frequency (LF) power or the ratio of LF/HF of HRV as the signs of high sympathetic tone remains after acute stress, that person is exposed to cardiac failure and arrhythmia [18] that should be evaluated.

According to previous results, the dynamics of biological signals also has a complex and chaotic pattern [15] and the non-linear analysis of biological signals can be considered as a highly reliable measurement [23]. Therefore, the aim of this study was to evaluate the persistent effect of acute stress, TSST, on emotional self-reporting, salivary cortisol, and linear and non-linear features of HRV in both genders. The hypothesis of the study was the fact that some features of HRV like cortisol made a persistent change after stress, although there were some sex differences in responding to stress.

## Material and methods

### Participants

Thirty-three young participants (23 men, 10 women) aged between 18 and 30 years entered the study. The inclusion criteria included general physical and mental health, no smoking habit, no surgery in the spine and cervicocephaly, no regular neuropsychological medication usage, no regular exercise, and no abnormal sleep pattern. Moreover, all of the participants signed the ethical consent approved by Baqiyatallah University of Medical Science.

### Procedure

**Fig. 1:**
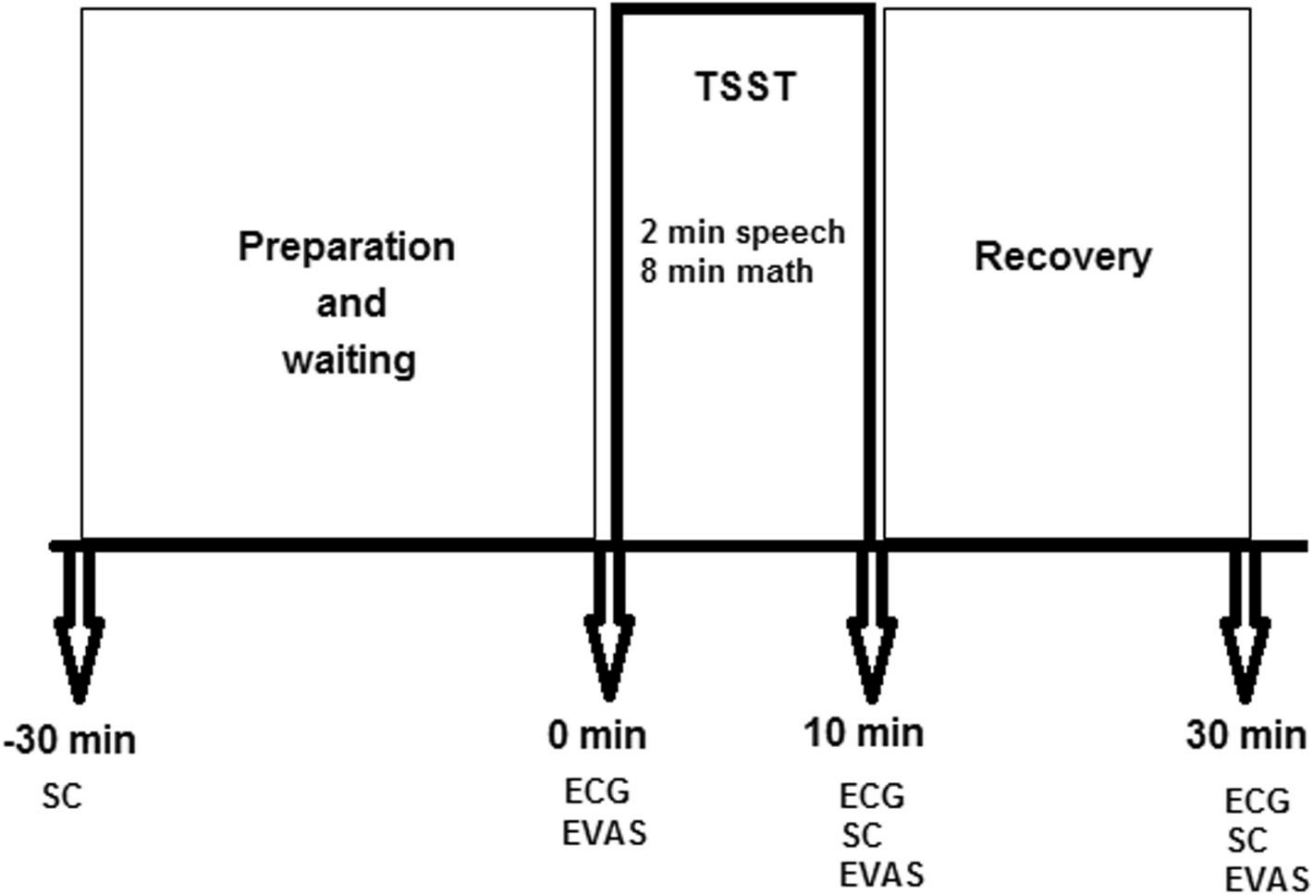
The time table of the study

The participants filled up the demographic characteristics, emotional intelligence questionnaire, and DASS 21 items. Since the level of cortisol and HRV is influenced by circadian rhythm, physical activity, and medication [7, 22, 34], subjects were tested between 10 PM and 2 AM in the same room at 23–25 °C. After asking about the drug consumption, those participants with no history of drug consumption were permitted to do the test. They were also asked not to eat anything 1 h before the test and wash their mouth carefully. The tests were done by taking the salivary sample, EVAS score, and ECG in three times (before TSST, after TSST, and 20 min after recovery) (Fig. 1). The TSST protocol consisted of an interview and an arithmetic task including a 5-min speech preparation period before coming to the test room, 2-min speech performance against two managers, and 8-min math portion. The participants were then asked to sequentially subtract number 13 from 1022 to the end. Examiners verbally reported their answers aloud and said to start over from 1022. If the participant had made a mistake, the examiners would have prompted them with: “That is incorrect, please start over from 1022.” [19]. The psychological measures on emotional visual analog scale (EVAS) consisted of a horizontal scaled line between 0 and 10 points. The subjects marked a point on the line resembling their subjective appraisal of stress perception, 0 = feeling good without distress to 10 = feeling highly and unbearable distress [12]. EVAS was asked in three sections of the study.

### Salivary cortisol

The saliva samples were collected minimally (0.5 mL) and frozen at −80. After thawing, they were mixed and centrifuged. The human saliva cortisol enzyme immunoassay (EIA) kit from IBL Company made in German was used. So, the procedure was done based on the kit’s instruction. The negative curve of TDS was determined, and then, the level of cortisol according to OD adjusted on the curve was obtained in each sample. The level of free cortisol of saliva was reported in nanomoles per liter. Reportable range of cortisol in this kit is 0.015–3 μg/dL, and the standard range mentioned in this salivary cortisol kit brochure is 0.9–9.2 nmol/L at 9–15 o’clock.

### ECG recording

The heart rate was concurrently recorded with EEG signal (not reported in this article) using Mitsar-EEG 202 Ac amplifier made in Russia. One previous study approved recording ECG signal by one channel simultaneously with EEG [17]. To this end, the active electrode was attached to the small finger of the left hand. The reference electrode was put on the center of the scalp in Cz position of EEG montage, and the earth electrode was in Fz position. Records were made for 2 min at each section. The subjects stayed in a sitting position without deep breathing or speaking during the test. Data was saved for 2 min in each section and relayed to an analog-to-digital converter in the sampling rate of 256 Hz. The HRV was analyzed offline using MATLAB software and HRV analysis codes. The linear features extracted from RR series in the time domain were mean and SD of RR. In the frequency domain, HF power (0.15–0.5 Hz), LF power (0.05–0.15 Hz), very LF power (0–0.04 Hz), and finally the ratio of LF/HF components were analyzed [1]. The non-linear features extracted in the time domain were SD1 and SD2 of Poincaré Plot [3] and the alpha 1 of detrended fluctuation analysis (DFA) [24], and in the frequency domain was spectral entropy (SpeEn) [28].

### Statistics

The *t* test compared the baseline characteristics and mental state such as age, level of cortisol, EQ, and DASS scores between genders, because they had normal distribution. Chi-square compared the percentage of education level between genders. Two-way mixed model ANOVA analyzed the interaction effect of gender and change of cortisol level and EVAS in three times of measurement (rest, stress condition, and post-recovery). Repeated measurement ANOVA was run to compare the level of cortisol, EVAS, and HRV linear features at three times of measurement in two groups of men and women separately. To compare the non-linear features in three times of measurement, the Friedman and Wilcoxon test was run. Less than 0.05 was considered as significant.

## Results

Twenty-three men and ten women participated in the present study. Table 1 shows the mean, the SD (except for the percentage of education level), and the comparison of the demographic and baseline of mental state in two genders. The results showed that there was no significant difference between the EQ and DASS score. The level of the baseline salivary cortisol and EVAS of the women was significantly higher than men, and the women’s education level was also higher.

**Table 1:** The comparison of demographic and psychological characteristics and cortisol level between two genders

	Men (*N* = 23)	Women (*N* = 12)	*p* value
Age (years)	23.37 ± 2.7	25.58 ± 4.6	0.14
DASS-depression	10.35 ± 10.5	9.64 ± 7.2	0.84
DASS-anxiety	7.91 ± 7.4	12.73 ± 8.7	0.1
DASS-stress	17.37 ± 10.5	15.82 ± 8	0.66
EQ	327.09 ± 35	336.3 ± 35	0.47
Education (percentage of diploma/licensed/graduated)	43.5/56.5	8.3/25/66.7	*0.00004*
Marriage status (percentage of yes/no)	13/87	75/25	*0.0004*
Rest condition cortisol (nmol/L)	3.03 ± 2	15.71 ± 10	*0.001*
Rest condition EVAS	1.1 ± 1.2	2 ± 1	*0.05*

Data is mean ± SD. Significant difference is shown as italicized data

The results of cortisol, psychological self-reporting of EVAS, and linear and non-linear HRV analysis have been brought in order.

### The EVAS score and salivary cortisol

**Fig. 2:**
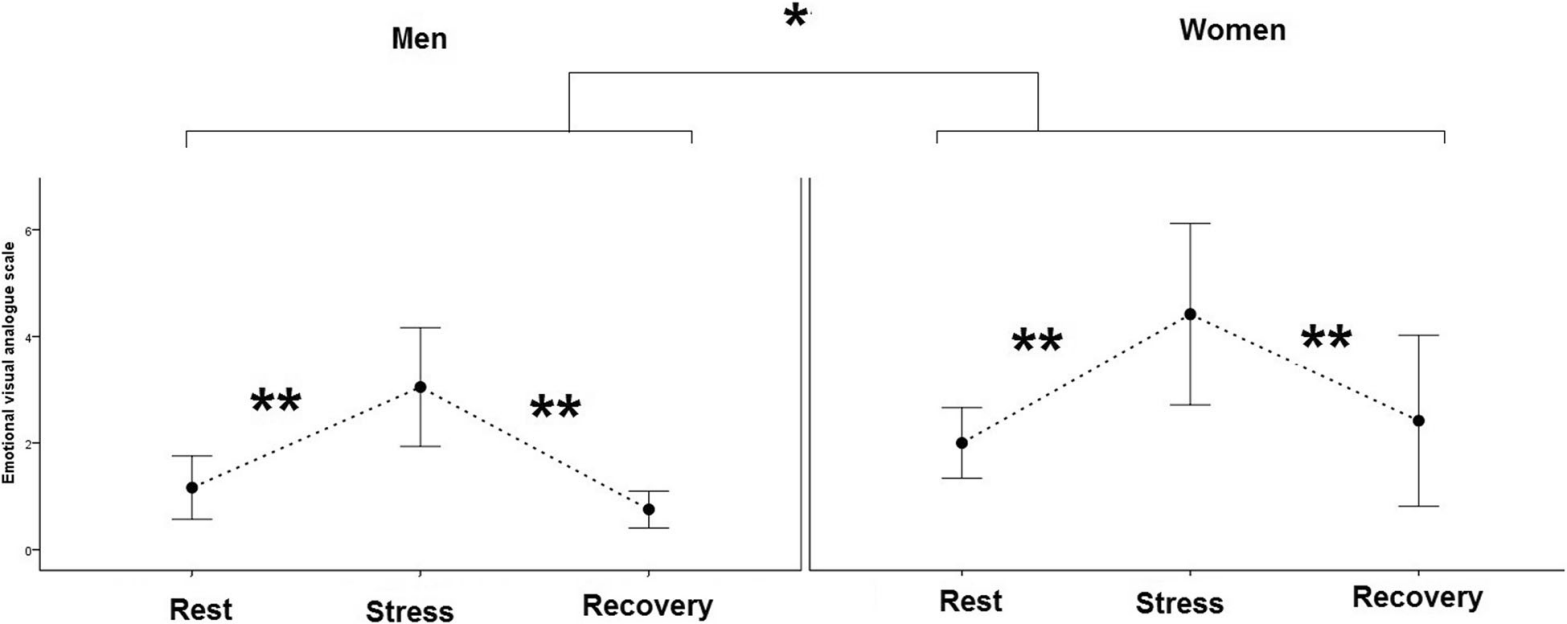
The plot shows the mean and CI 95% of emotional visual analog scale in the men and women in the rest, stress, and recovery condition. *p < 0.05, **p < 0.00001

**Fig. 3:**
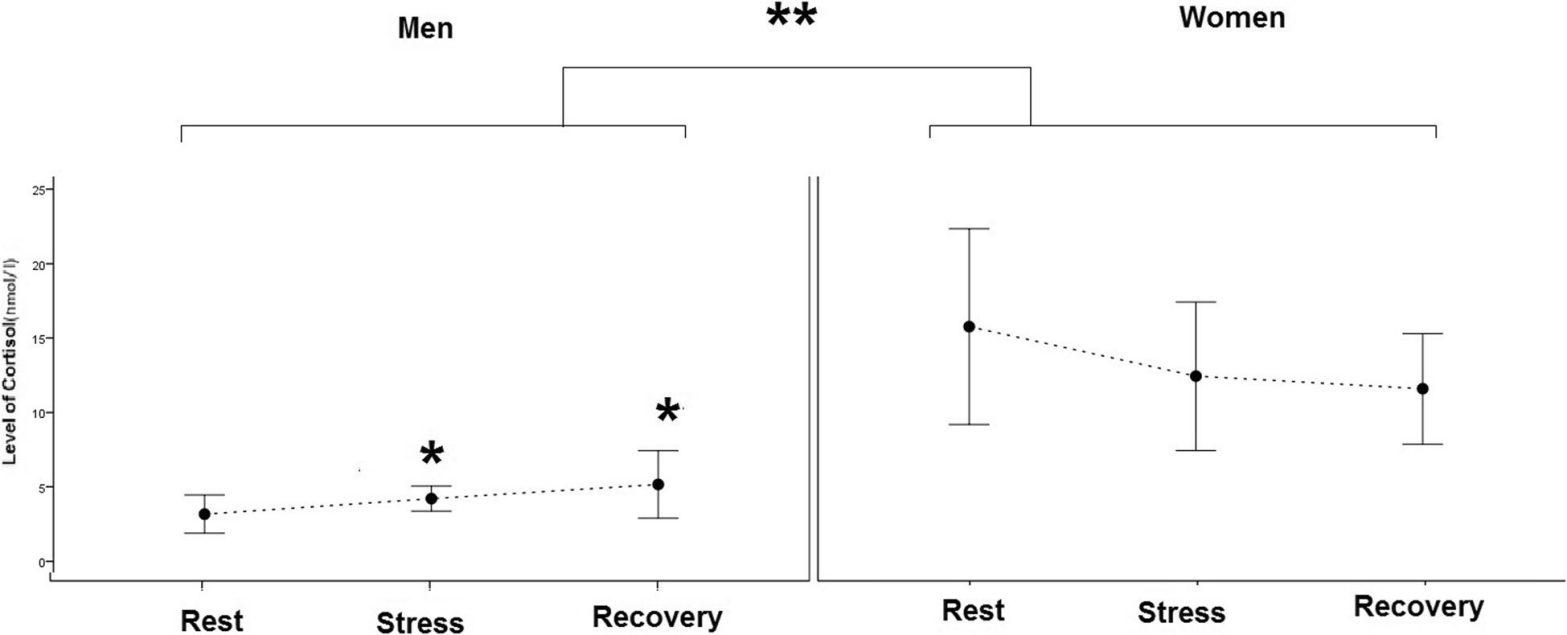
The plot shows the mean and CI 95% of salivary cortisol level in the men and women in the rest, stress, and recovery condition. *p < 0.05 for comparison between prestress with other stages in men. **p < 0.00001 for comparison between men and women

The two-way mixed model ANOVA showed that TSST protocol increased the negative mood in all participants based on the EVAS score. The EVAS score returned to the baseline state 20 min after recovery (*F* (1, 34) = 21.13, *p* < .0.000002). The women also showed more EVAS score in comparison with the men (*F* (1, 34) = 6.39, *p* < 0.02) (Fig. 2). Although the level of cortisol in the women was higher than in men (*F* (1,34) = 23.1, *p* value < 0.00001), the level of cortisol in the stress condition significantly increased only in men (*F*(1,34) = 4.28, *p* value < 0.05) (Fig. 3).

### The linear features of HRV

**Fig. 4:**
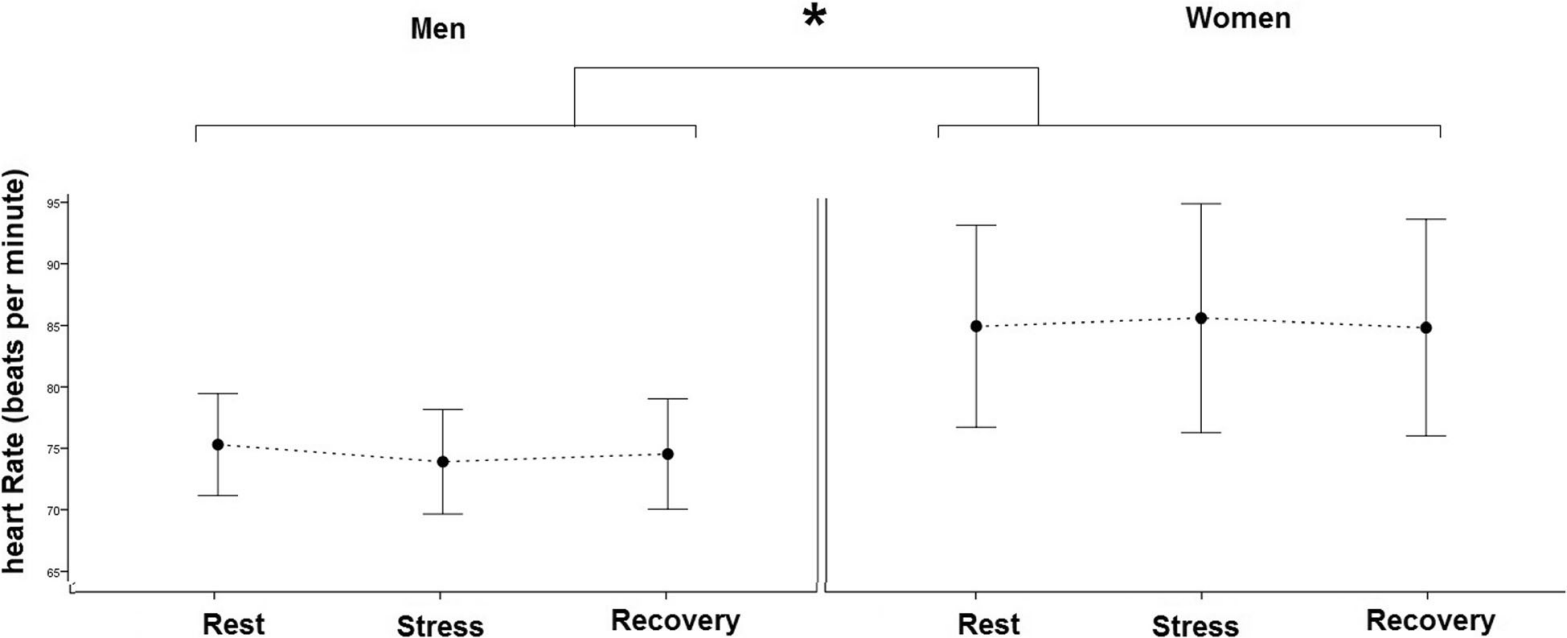
The mean and CI 95% of heart rate of the men and women in three times of the test. *p < 0.05 between men and women

The two-way mixed model ANOVA test showed that there was no significant difference between three times of heart rate recording, time between R-R peaks, and its SD in both genders. However, there was a difference between genders (*p* < 0.05). It means that the women had more heart rate (Fig. 4) and less RR interval and its SD (*p* < 0.05).

**Fig. 5:**
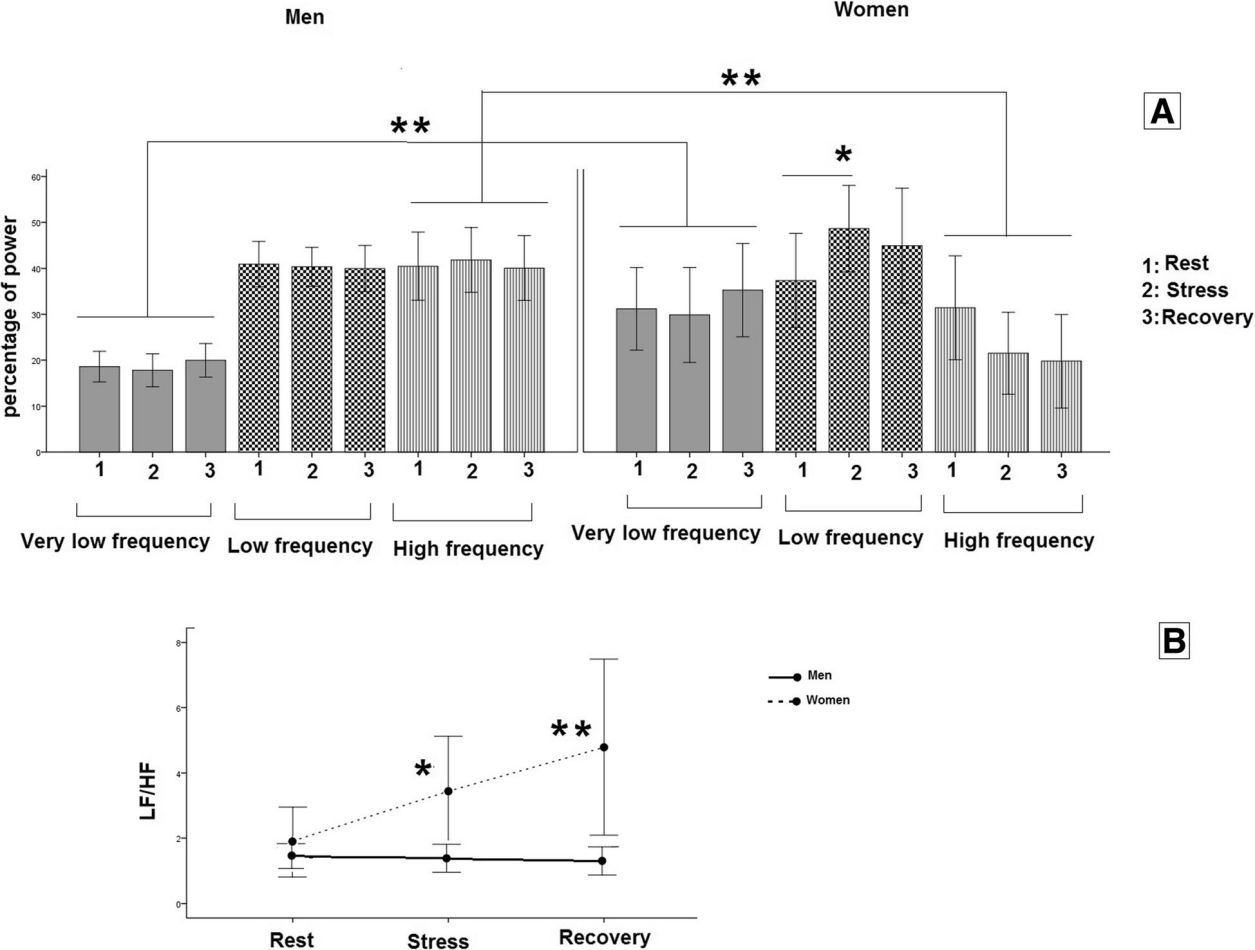
The mean and CI 95% of the percentage of HRV (**a**) power frequency and ratio of LF/HF (**b**) in the men and women, in three phases of the test

Figure 5 shows that the percentage of power frequency changed after stress just in the women. The results of 2-way mixed model ANOVA showed that the percentage of LF and LF/HF ratio significantly increased only in the women (LF: *p* < 0.001, LF/HF: *p* < 0.0001). The percentage of HF decreased after stress in the women but not significantly. In general, the percentage of VLF was significantly more in the women and HF was significantly more in the men (*p* < 0.005).

### The non- linear features of HRV

**Fig. 6:**
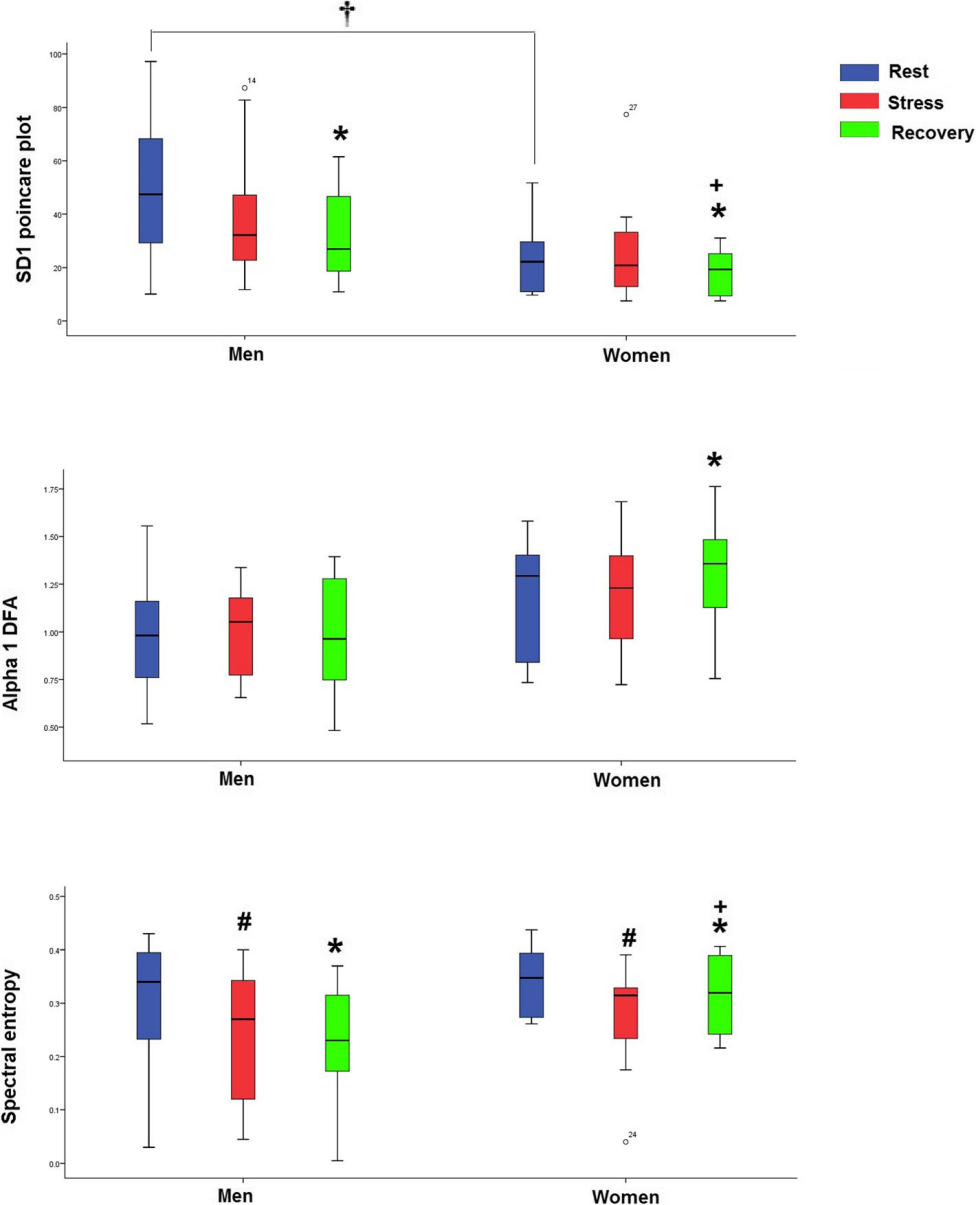
The box plot of SD1 Poincaré, alpha 1 of DFA, and spectral entropy in the men and women and in three phases of the test. *, the significance between rest and recovery; #, the significance between rest and stress condition; +, the significance between stress condition and recovery; †, the significance between men and women. Alpha 1 of DFA index increased after stress and recovery only in women (between rest and recovery: *Z* of Wilcoxon’s test = − 2. 9, *p* < 0.004; between stress and recovery: *Z* of Wilcoxon’s test = − 2. 27, *p* < 0.02). There was no significant difference between genders

Figure 6 shows the non-linear features of HRV, SD1 of Poincaré, alpha 1 of DFA, and spectral entropy. The SD1 of Poincaré plot decreased after recovery to compare the rest condition in both of the men (*Z* of Wilcoxon’s test = − 2.76, *p* < 0.006) and women (*Z* of Wilcoxon’s test = − 2.51, *p* < 0.01). The sex difference of SD1 of Poincaré was observed in the rest condition (*Z* of the Mann-Whitney test = − 3.14, *p* < 0.001). The SD2 of Poincaré did not significantly change after TSST.

Spectral entropy significantly decreased after stress in the men (between rest and stress condition: Z of Wilcoxon’s test = − 2.46, *p* value < 0.014; between rest and recovery: *Z* of Wilcoxon’s test = − 2.33, *p* < 0.02) and increased in the women (between rest and stress condition − 1.88, *p* < 0.06; between rest and recovery condition: *Z* of Wilcoxon’s test = − 2.59, *p* < 0.01).

Considering all the cases, there was a high correlation between the baseline level of cortisol and change of cortisol after TSST (Pearson’s correlation = − 0.82, Sig = 0.000000, *N* = 98) and no correlation between baseline cortisol and EVAS before TSST. Then, the cubic non-linear relation was considered significant between cortisol level and some features of HRV. The regression between cortisol and heart rate, percentage of VL power and α1 of DFA was positive, while the regression between cortisol and othervariables was negative (Table 2).

**Table 2:** The *R*^2^ and significance of cubic regression between the level of salivary cortisol and HRV features in the 98 tests

		RR	SD of RR	HR	PVLF	PHF	SD1 of PP	α1 of DFA
Cortisol	*R* ^2^	0.225	0.129	0.26	0.29	0.162	0.194	0.13
	Sig	0.000	0.008	0.000	0.000	0.002	0.000	0.008

*RR* time between beats, *HR* heart rate, *PVLF* percentage of very low frequency to all frequency, *PP* Poincaré plot, *DFA* detrended fluctuation analysis

There was a significant positive linear correlation between the ratio of LF/HF and SD2LSD1 of Poincaré plot (*R* = 0.73, *p* < 0.0000001).

## Discussion

The aim of the study was to compare genders in terms of the persistent response to stress based on the self-report, EVAS, salivary cortisol, and HRV linear or non-linear indices. The EVAS score was significantly increased in both genders. Although the level of cortisol measured before TSST was around 4 times higher in the women in comparison with men, it did not increase in the women after stress. On the other hand, the increase of sympathetic tone based on the power frequency of HRV and LF/HF ratio was only observed in the women after stress, which remained 20 min after recovery. In addition, the results revealed a high negative significant correlation between the baseline cortisol and the increase of cortisol level after TSST. The significant correlation was also seen between the level of cortisol and some features of HRV. Thus, it can be concluded that an effective factor in changing cortisol after TSST is the rest cortisol level before TSST that should be more than the mentioned standard range of the salivary cortisol of the kit (0.9–9.2 nmol/L at 9–15 o’clock) [16] not gender [10]. Previous cohort study also showed that men and women with acute heart failure had different baseline clinical characteristics but not primary etiology of heart failure [11]. Moreover, the absolute cortisol did not correlate with emotional self-reporting of distress or state anxiety [30, 31]. These results were observed in the young people and might not be observed in other age ranges.

Several studies demonstrated that the level of cortisol, perceived stress, and anxiety score increased during and immediately after TSST, but the increased heart rate was only observed during TSST [2, 12, 19, 34]. Several factors from some diseases of innervations have also influence on the cortisol elevation due to acute stress or TSST [8]. The women who were under stress or in the luteal phase showed less elevation of cortisol due to TSST [8, 32]. Besides that, the procedure of the test might affect the result. In fact, the preparation time for TSST, the content of the speech task, and the matching of participants and managers regarding the gender would change the response to stress as well [10]. When the participants and managers were of the same gender, the increase of cortisol was lesser. On the other hand, some studies demonstrated that a person was a responder to stress with the increase of cortisol whenever others of both genders were not responder [27]. These studies revealed that the non-responder had more activity in the ventromedial prefrontal cortex (mPFC) and posterior cingulate cortex. According to these studies, the more activity of mPFC integrated emotional reporting and endocrine response to stress [36]. Thus, in the current study, the fact that women were non-responding to stress in terms of cortisol was related to several factors as mentioned above [29]. In addition, prestress cortisol level of the women was 4 times more than men and the elevation of cortisol after stress had a high negative correlation with the baseline cortisol. Besides that, the base of cortisol was specifically more than the standard range mentioned above which is a very predictable factor of cortisol elevation due to acute stress.

The persistence of biological changes due to stress is critical because the overload of stress is dangerous for hemostasis and returning to the normal state. Recovery time after TSST was evaluated to reach the required interval time between stressful status to prevent allostatic load and overload [21]. Previous studies showed that the increase of cortisol continued until 15–20 min and returned to the baseline 40–60 min after TSST [12, 32]. The dynamic shifts in the brain networks to comprehensively reallocate its neural resources against stressful condition according to cognitive demands was reverse to prestress about 60 min [13]. But some other markers such as salivary alpha-amylase enzyme or heart rate and RR mean of HRV immediately returned to the baseline after TSST [20].

The findings of the current study regarding HRV showed that the heart rate and mean of RR interval returned to the baseline state immediately after stress, as the previous studies have confirmed it [19, 32, 34, 36]. There are many studies showing that the power of frequency or non-linear features of HRV change during acute stress as the sign of the increased sympathetic tone activity in both genders [4, 33]. In addition, it has been proven that the LF and LF/HF mostly increased and the complexity of HRV decreased based on SD of RR and Poincaré plot, sample and spectral entropy, and alpha 1 of DFA during stress, which are also the signs of sympathovagal shift to sympathetic tone [25, 27, 33]. A positive regression between the ratio of LF/HF and ratio of SD2/SD1 of Poincaré plot approved by previous studies showed that the LF of spectral analysis of HRV increases and the SD1 of Poincaré plot decreases in the sympathetic dominance [3, 9]. Our data also confirmed this regression. However, there is no study assessing the persistence of these features of HRV after stress or recovery.

The non-linear features provide more complete and natural information about the biological system [5]. The cardiovascular system fluctuates between a set of metastable states to adapt to internal or external challenges in normal conditions. This fluctuation is a result of complex oscillators that work together such as the circulation of blood, respiratory, and reticular rhythm (autonomic control origins from brainstem reticular formation) [35]. The frequencies of these rhythms and the strength of their coupling change in the course of time depend on a wide range of physical or mental state such as stress. If the cardiovascular fluctuations change toward more regular and periodic behavior, the coupling and phase integration of oscillators becomes weak. Then, the person is prone to cardiovascular impairment [14]. Here, the results showed that the complexity of HRV based on spectral entropy and SD of RR and Poincaré plot decreased after stress and, even, recovery and there was a negative correlation between the level of cortisol and amount of these indices. The increase of the percentage of LF and the ratio of LF/HF did not also return to a normal state in women after recovery. These results can be taken into account as a kind of warning to public health, since the remained reduction in heart rate complexity after stress may represent a lower adaptability and a functional restriction of the participating cardiovascular elements.

The limitations of the present study were the small number of the participating women compared with men and not using the respiratory assessment concurrently with ECG to well evaluate the power frequency of HRV. Moreover, the ECG recording was not saved during TSST. In addition, the stress intensity of TSST in our study was not enough to produce high changes in both genders.

TSST as an acute stressor caused some persistent effects on hormonal factor, frequency, and complexity features of HRV in both genders. The physiological response to acute stress was correlated with the baseline cortisol, independent of the gender. The baseline cortisol of the women was 4 times higher than men and higher than the standard range as well. In addition, the sympathetic response to stress in the women was higher than men, although their cortisol level did not elevate after TSST. Some non-linear features of HRV such as SD1 of Poincaré plot and spectral entropy decreased in both genders until 20 min after recovery. Although the self-reporting showed that nobody felt stress after recovery, the physiological assessment revealed some physiological changes due to stress that even remained after recovery.

## Data Availability

The datasets used and/or analyzed during the current study are available from the corresponding author on reasonable request.

## Abbreviations

DASS: Depression anxiety stress score
DFA: Detrended fluctuation analysis
ECG: Electrocardiography
EEG: Electroencephalograpy
EIA: Enzyme immunoassay
EQ: Emotional intelligence
EVAS: Emotional visual analog scale
HF: High frequency
HPA: Hypothalamus-pituitary-adrenal
HR: Heart rate
LF: Low frequency
mPFC: Medial prefrontal cortex
SAM: Sympathetic-adrenal medullary
SD: Standard deviation
SpeEn: Spectral entropy
TSST: Trier Social Stress Test
VLF: Very low frequency
